# Maffucci Syndrome

**Published:** 2014-04-25

**Authors:** Joshua B. Elston, Wyatt G. Payne

**Affiliations:** Plastic Surgery Section, Bay Pines VA Healthcare System, Bay Pines, Fla; and the Division of Plastic Surgery, Department of Surgery, University of South Florida College of Medicine, Tampa, Fla

**Keywords:** Maffucci syndrome, enchondroma, hemangioma, venous malformations, spindle cell hemangioma

## DESCRIPTION

A 13-year-old girl presented with swelling and tenderness of the digits of the hand. She had undergone prior excision of similar lesions. These lesions have been present since approximately the age of 4, are bilateral, and have become progressively troublesome and painful. She was noted to have an asymmetric gait and unequal extremity lengths.

## QUESTIONS

1. **What is the typical age at which onset of symptoms occurs?**
2. **What is the risk of malignant degeneration of the bony lesions?**
3. **What are characteristic features seen on hand x-ray in this disease?**
4. **What is the pattern of inheritance of Maffucci syndrome?**

## DISCUSSION

Maffucci syndrome typically begins to manifest around 4 to 5 years of age and progresses throughout life.^1^ The key clinical features of this disabling disease are multiple enchondromas—typically of the distal extremities—and venous malformations with spindle cell hemangiomas that can develop anywhere on the skin.^1^ The vascular lesions in Maffucci syndrome appear as reddish-blue, soft, compressible, occasionally tender, subcutaneous nodules. Most reports describe acral manifestations; however, there have been reports describing lesions of the gastrointestinal system and the upper airways. Involvement of the gastrointestinal tract can result in occult microcytic anemia from hemorrhage, whereas lesions in the upper airways can rupture and cause rapid respiratory compromise.^2^^,^^3^ Symptoms can range from local soft tissue swelling causing cosmetically unsightly extremities to painful lesions that are at risk for hemorrhage if traumatized.

It is important to recognize the diagnosis and differentiate Maffucci syndrome from the clinically similar Blue Rubber Bleb Nevus (BRBN) syndrome because of the significant risk for malignant degeneration of enchondromata.^4^ The differentiating factor is that in BRBN syndrome, there will be no bony involvement. Oftentimes, these enchondromata seen in Maffucci syndrome can cause pathologic fractures that lead to bony deformation, shortening, and difficulty in manipulating objects or ambulating. Serial radiographs looking for cortical destruction, endosteal cortical erosion, and zones of lucency within a previously mineralized area should be performed to monitor for the development of chondrosarcomas—a 30% risk.^5^

The characteristic features of Maffucci syndrome seen on hand x-ray are the multiple acral enchondromata that progress over time. These radiographs should be followed by a Hand or Orthopedic surgeon to assess for malignant degeneration. Because of the multiple spindle cell hemangiomas present in the hands, characteristic calcific phleboliths may also be visualized within the vascular malformations.

Maffucci syndrome is a rare, sporadically inherited genetic disorder that does not display any sexual or racial predilections. Because of the rarity of the disease, specific genetic markers have long eluded association with this syndrome. However, a link with somatic mosaic mutations in the isocitrate dehydrogenase 1 and 2 (IDH1 and 2) genes have been associated with both Maffucci syndrome and Ollier disease. A recent study found that roughly 80% of patients studied with both Ollier disease and Maffucci syndrome had heterozygous somatic mutations of the IDH1 or 2 genes in their tumors.^6^

Maffucci syndrome had long been a clinical diagnosis. There is no hereditary transmission and no etiological predilections. Recently, a link with mutations in the IDH1 and 2 genes has been associated with this process. It is important to distinguish this syndrome from BRBN syndrome due to the roughly 30% risk for malignant degeneration of the cartilaginous lesions over time into chondrosarcomas. Symptoms of this syndrome may range from asymptomatic soft tissue swelling to painful lesions that hemorrhage causing anemia or pathologic fractures that deform the bony skeleton and impair function.

## Figures and Tables

**Figure 1:**
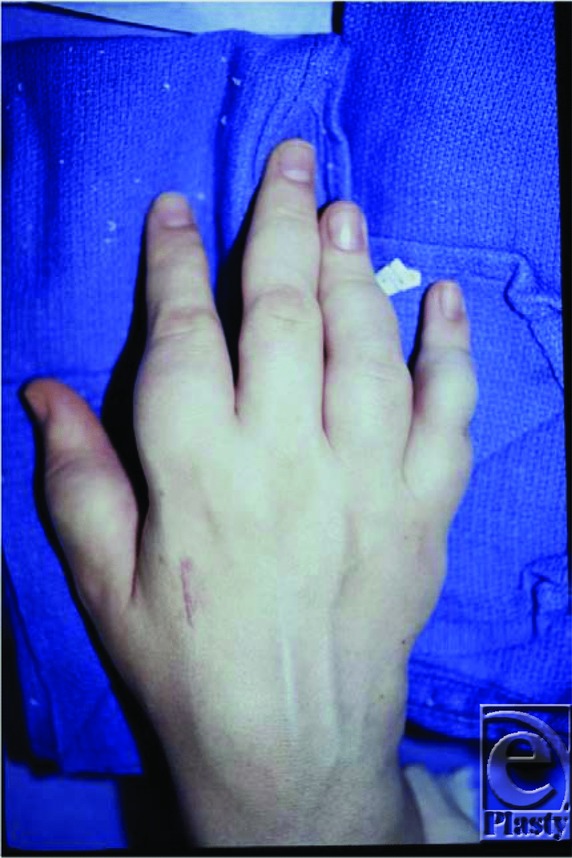
Multiple hemangiomas causing painful and unsightly soft tissue swelling.

**Figure 2:**
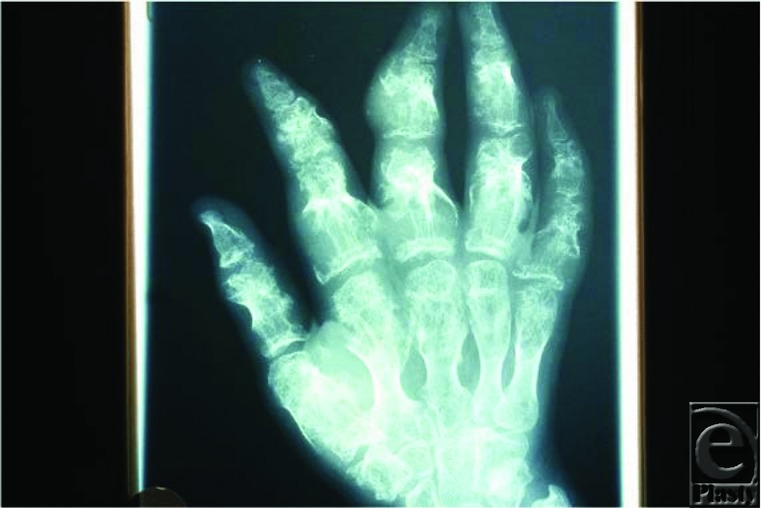
Radiograph demonstrating multiple enchondromas and bony deformation.

**Figure 3:**
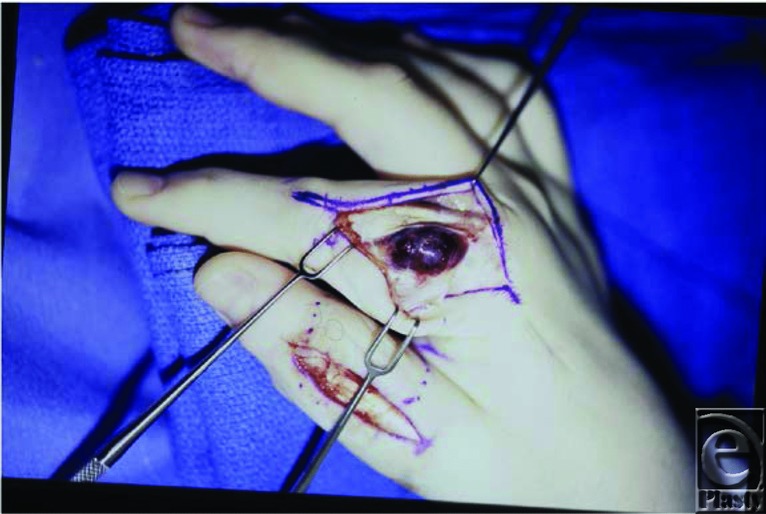
Surgical excision of digital hemangioma.
